# Medical-grade Sterilizable Target for Fluid-immersed Fetoscope Optical Distortion Calibration

**DOI:** 10.3791/55298

**Published:** 2017-02-23

**Authors:** Daniil I. Nikitichev, Dzhoshkun I. Shakir, François Chadebecq, Marcel Tella, Jan Deprest, Danail Stoyanov, Sébastien Ourselin, Tom Vercauteren

**Affiliations:** ^1^Translational Imaging Group, CMIC, University College London; ^2^Department of Obstetrics and Gynecology, University Hospitals Leuven; ^3^Surgical Robot Vision Group, CMIC, University College London

**Keywords:** Bioengineering, Issue 120, fluid-immersed optical distortion calibration, endoscopy, prototyping, medical device, autoclaving, medical imaging

## Abstract

We have developed a calibration target for use with fluid-immersed endoscopes within the context of the GIFT-Surg (Guided Instrumentation for Fetal Therapy and Surgery) project. One of the aims of this project is to engineer novel, real-time image processing methods for intra-operative use in the treatment of congenital birth defects, such as spina bifida and the twin-to-twin transfusion syndrome. The developed target allows for the sterility-preserving optical distortion calibration of endoscopes within a few minutes. Good optical distortion calibration and compensation are important for mitigating undesirable effects like radial distortions, which not only hamper accurate imaging using existing endoscopic technology during fetal surgery, but also make acquired images less suitable for potentially very useful image computing applications, like real-time mosaicing. In this paper proposes a novel fabrication method to create an affordable, sterilizable calibration target suitable for use in a clinical setup. This method involves etching a calibration pattern by laser cutting a sandblasted stainless steel sheet. This target was validated using the camera calibration module provided by OpenCV, a state-of-the-art software library popular in the computer vision community.

**Figure:**
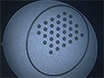


## Introduction

Camera calibration is a well-known problem in the computer vision field that has been intensively studied over the years^1^,^2^,^3^. A key step of camera calibration procedures is to estimate the parameters of a distortion model, as well as the intrinsic camera parameters, by extracting a grid of points with a known geometry from camera images with sub-pixel accuracy. Calibration targets with a checkerboard pattern featuring black and white squares are commonly used for this purpose. Circular blobs offer an alternative pattern^4^,^5^,^6^.

In recent years, there has been a growing interest in the development of surgical navigation technology for fetal surgery procedures, such as the treatment of twin-to-twin transfusion syndrome (TTTS) on fetuses^7^,^8^,^9^,^10^. As the field of view of the fetoscope (*i.e.,* an endoscope used in fetal surgical procedures) is very limited, methods for mapping the placental vasculature without the use of external trackers have been proposed to aid TTTS surgery^11^,^12^,^13^. Optical distortions within fetoscopic images have adverse effects on these computational mosaicing methods that rely on visual information extraction^11^. Thus, there is an unmet need for a cost-efficient and fast tool for peri-operatively calibrating fetoscopes so that optical distortion compensation can be done in real-time during the intervention.

Due to the fact that the fetoscope is immersed in amniotic fluid during the intervention, the refraction index difference between air and amniotic fluid renders classical in-air camera calibration methods unsuitable for fetal surgery procedures. Estimating fluid-immersed camera parameters from in-air camera parameters is a difficult task and requires at least one image of the fluid-immersed calibration target^14^. Furthermore, peri-operative, fluid-immersed fetoscopic camera calibration is currently impractical due to sterilization requirements and restrictions on the materials allowed in the operating theater. Due to these reasons, calibrating endoscopes for optical distortions is typically not part of the current clinical workflow. The work in this manuscript is an attempt to close this camera calibration gap by designing and producing a sterilizable and practical optical distortion calibration target featuring a pattern of asymmetric circles. Previously, Wengert *et al.* fabricated a custom calibration device featuring an oxidized aluminum plate as the calibration target. Their method, however, works only in conjunction with the custom calibration algorithm they developed^15^.

## Protocol

### 1. Target Fabrication

1. Sandblasting
  1. Prepare a 316 stainless steel sheet with a 1.2-mm thickness. Using a pencil or a nail, draw a 40 mm x 40 mm square onto the sheet with the aid of a ruler.
  2. Cut the drawn square using a manual metal cutter. CAUTION! Watch the fingers.
  3. Use a file to round the corners and sides of the sample. CAUTION! They are very sharp; be careful.
  4. Prepare a straight wooden or metal block slightly larger in size than the stainless steel sheet. Place the cut sheet on it; do this in order to avoid bending the sample during sandblasting.
  5. Place the assembly in the internal blast chamber. Remember to use a dust collector and to tightly seal the internal blast chamber; otherwise, the sand will spread all over during the process. Wear safety goggles to protect the eyes.
  6. Position a blast gun perpendicular to and at least 4-5 cm away from the metal surface. Apply the foot control for sandblasting. Put the sample on the piece of wood (1-2 cm thick) using a vice, as the high-pressure sand flow can deform the sample. During sand-blasting, hold the sample tightly by the edge of the piece of wood or by using another vice.
  7. Repeat the sandblasting on the other side if it is desirable to have a calibration pattern engraved on both sides.
2. Laser patterning
  1. Design a pattern of asymmetric circles, as shown in **Figure 1**.
  2. Prepare a drawing exchange format (DXF) file of the design either using CAD software or another suitable programming language. NOTE: For convenience, a Python application that can generate DXF files for the design mentioned in this paper is provided as part of the compact GUI application^16^.
  3. Import the DXF files into the laser cutting software.
  4. Set up the following parameters for background etching. Laser Power: 40%, Scan Speed: 80 cm/s, Frequency: 4,000 Hz, Number of Passes: 1.
  5. Set up the following parameters for etching the pattern. Laser Power: 40%, Scan Speed: 2.1 cm/s, Frequency: 4,000 Hz, Number of Passes: 1.
  6. Put the sample on the working platform and align the cutting pattern using the software.
  7. After the laser performs the cut, clean the sample by dipping it in alcohol. Do not use any wipes, as they usually leave undesirable residue.
3. Sterilization
  1. Wrap the sterilized sample in a sterilization package and insert it in the sterilization unit (autoclave).
  2. Add water (not distilled water) to the autoclave and follow the user's guide/manufacturer's recommendations to sterilize the target.

### 2. Peri-operative Calibration

1. Calibration software
  1. Install the "endocal" endoscope calibration software package provided on GitHub^16^ (follow the instructions in the README file therein). NOTE: This software wraps the OpenCV camera calibration module^17^ in an easy-to-use convenience application. The provided application runs in two modes: online and offline. The online mode acquires the video stream directly from compatible frame-grabber hardware. The offline mode allows for loading endoscope images either from a video file or a folder with a number of video frames saved as image files. See README for supported hardware and detailed instructions on how to use these two modes.
2. Endoscopic video acquisition NOTE: The following instructions are for online calibration (as described above), but they are also applicable to offline calibration.
  1. Place the calibration target in a sterile fluid container, such as a gallipot.
  2. Fill the container with the target fluid or a similar sterile substance. NOTE: For instance, in fetoscopic procedures, the target fluid is amniotic fluid. Since the optical properties of amniotic fluid are similar to saline water^18^,^19^, sterile saline water can be used for calibrating the fetoscope.
  3. Adjust the zoom and sharpness of the endoscope as desired.
  4. Immerse the endoscope in the fluid and hold it at a distance from the calibration target similar to the distance from the anatomy that the endoscope will later be used at.
  5. Launch the calibration application and start the camera acquisition.
  6. Move the tip of the endoscope slightly for different views while keeping the whole calibration pattern in view of the camera. For optimal performance, keep the elliptical legend around the calibration pattern within the circular view of the endoscope. NOTE: Video frames that are usable for calibration are indicated by a virtual pattern overlay, as seen in **Figure 3**.
  7. Acquire at least the minimum number of endoscopic camera views required for calibration (as indicated in the endocal window). NOTE: The current version of endocal requires at least 10 endoscopic camera views for calibration, a heuristically selected number of views where the calibration error appears to be minimal and follow a stable pattern^20^.
  8. Press the calibration key, as indicated on the endocal window, to start the calibration process using the images acquired so far.
3. Saving and using the calibration parameters
  1. Press the indicated calibration key to save the resulting calibration parameters in a YAML ("YAML Ain't Markup Language") file^21^.
  2. Group the calibration parameters into the camera matrix and distortion coefficients, as explained in the OpenCV camera calibration module^17^. NOTE: After performing the calibration, the calibration application automatically displays the distortion-corrected image to the right of the original endoscope image.
  3. Use the distortion-corrected video feed during a fetoscopic procedure for pure visualization or for real-time placental mosaicing^11^.

## Representative Results

We created a sterilizable calibration target by etching a pattern of asymmetric circle on a sandblasted stainless steel metal sheet, whose design is shown in **Figure 1**. An exemplar setup showing this calibration target in action together with a fetoscope is shown in **Figure 2**. To feed this design into the laser etching software, a custom application was implemented in the Python programming language^16^. Creating the design pattern involves iteratively etching parallel lines on a metal sheet. For the pattern to have a consistent color in the end, the distance between these lines should be less than the width of the laser beam (see the inset of **Figure 1**)—this value is 45 µm for the Violino (Laservall) laser cutter.


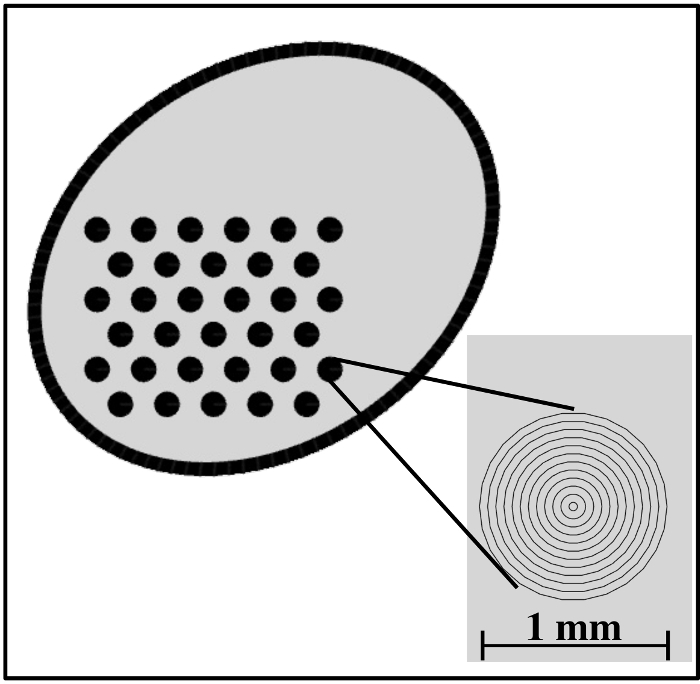
**Figure 1: Design of the engraved pattern featuring a 3-by-11 grid of asymmetric circles. **Inset: zoomed-in view of the grid of asymmetric circles. The distance between the lines is 45 µm (equal to the laser beam width), and each circle has a diameter of 1 mm. Other sizes could be used for the grid as well, but this was found to be optimal with respect to the fetoscope field of view. Please click here to view a larger version of this figure.


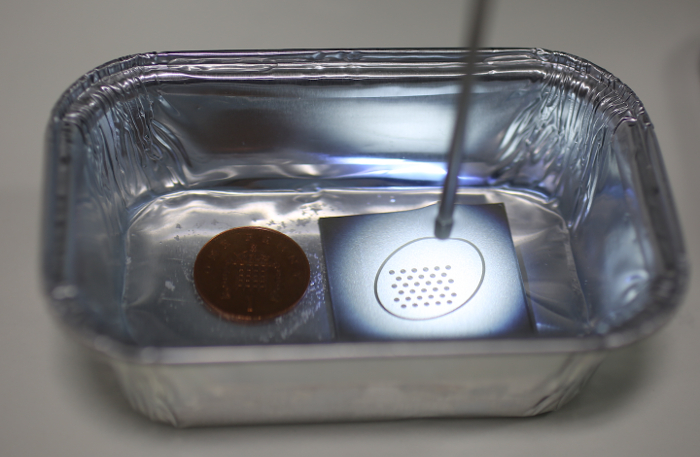
**Figure 2: Exemplar setup with the calibration target in use. **The tip of the water-immersed fetoscope is directed at the calibration target on the right. On the left is a British penny to provide scale information. Please click here to view a larger version of this figure.

The fabricated calibration target allows for the detection of the circular pattern in the endoscopic video stream with OpenCV^17^, whose locations are then sorted into the pre-defined asymmetric circular grid (see **Figure 3**). Using this information in conjunction with the already-known grid geometry, internal camera parameters can be estimated. These include the camera matrix and the distortion coefficients. The camera matrix consists of the focal lengths and the optical centers along the x- and y-axis of the 2D image plane. The distortion coefficients are based on the Brown-Conrady model^3^. Note that for this work, only the radial distortion parameters were estimated. For a brief discussion of the theory, with practical examples, see the webpage of the OpenCV camera calibration module^17^ and the MATLAB camera calibration toolbox^22^. More details about the camera calibration procedure are available in Zhang's work^20^. The endocal software repository features a sample dataset of 10 endoscopic views of the fabricated calibration target^16^. Using this dataset, a calibration with an average re-projection error of 0.28 pixels (min: 0.16, max: 0.45) was obtained. This is comparable to the 0.25 pixels reported by Wengert *et al.* using their custom calibration algorithm^15^. The same research group, however, reported a re-projection error of 0.6 pixels in a more recent paper when using the method in^15^ for calibrating an endoscopic camera used for placental mosaicing^18^.


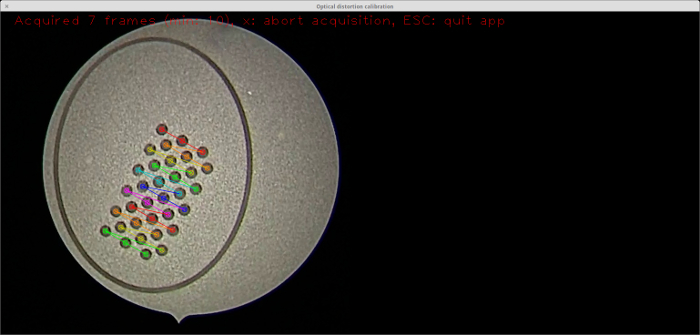
**Figure 3: Real-time detection of the calibration pattern. **A screenshot from the calibration application^16^ featuring the detected calibration pattern overlaid onto the live video stream using the virtual reality visualization from OpenCV^17^. Note that each detected column of the calibration pattern is emphasized by a different color. The detected circles, in conjunction with the known geometry, are used for computing the camera parameters. Please click here to view a larger version of this figure.

The estimated camera parameters are used for optical distortion correction. **Figure 4** shows a rectangular chessboard pattern, as viewed using a fetoscope, where optical distortions make the lines appear as curves. Note that the lines appear normal in the distortion-corrected image.


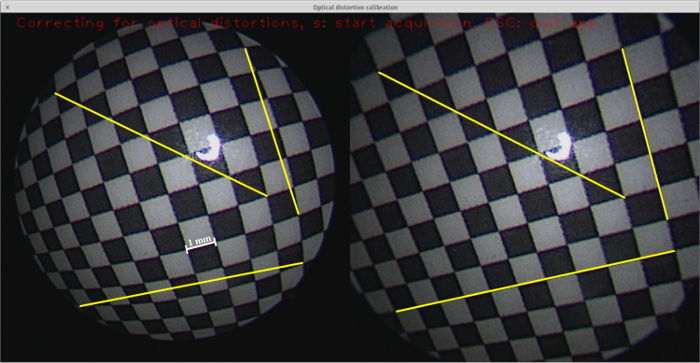
**Figure 4: Optical distortion correction. **A screenshot from the calibration application^16^ featuring the live video image from a fetoscope recording from the checkerboard pattern (left) with the distortion-corrected image (right). Three exemplary lines are drawn in both images, each from one corner to another, where the trajectory is linear. Due to the optical distortions, these lines appear as curves in the original fetoscope images. Please click here to view a larger version of this figure.

## Discussion

Sandblasting is an important step in the fabrication process because the raw metal surface prominently reflects the endoscope light, making it impossible for the circles to be detected. It is difficult to distinguish the circles even with the naked eye (see **Figure 5**). Note that the surface of the target shown was already etched with a laser. However, this does not diminish light reflection.


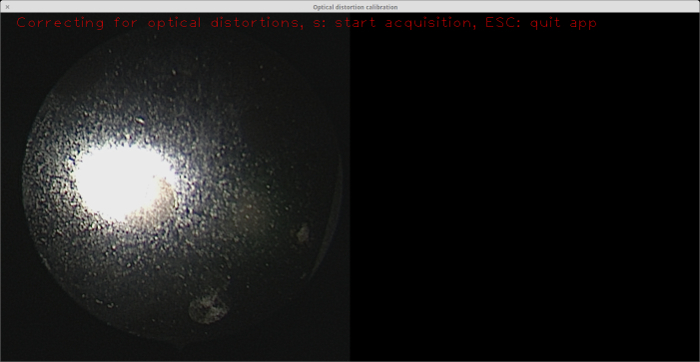
**Figure 5: Calibration target with no sandblasting applied. **As seen from the endoscope view on the left, the glare from the endoscope light on the material surface makes it difficult even for the naked eye to distinguish the circles (there is a circle just to the southeast of the large reflection). Note that the surface of this target (*i.e.,* the "background") was already etched, but this is not helpful in the absence of sandblasting. Please click here to view a larger version of this figure.

Prior to pattern etching, it is also important to etch the surface of the whole sample. This is necessary because the sandblasted surface has many specular reflections (see **Figure 6**), which interfere with blob detection.


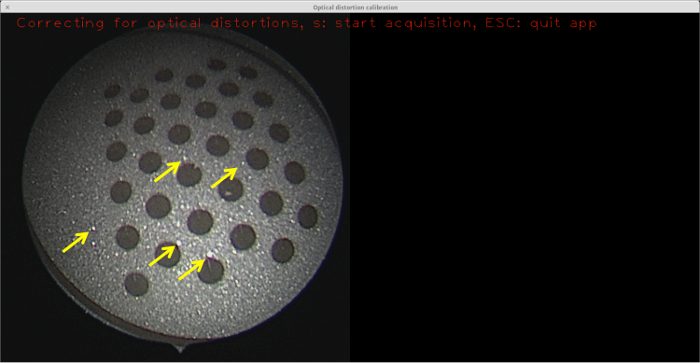
**Figure 6: Sandblasted surface with no etching. **Although not as prominent as the raw metal surface, the relatively small specular reflections (some of which are highlighted with yellow arrows) are still sufficient to prevent blob detection from succeeding, so no calibration can be performed with this target. Please click here to view a larger version of this figure.

Applying the laser at different speeds gives different background colors. The background color plays a significant role in the contrast between the circles and the background. Hence, it is vital to determine the optimal background color. For this purpose, a plate with circles etched against a set of different backgrounds was created (see **Figure 7**). The backgrounds were tested using the feature detection module of OpenCV^23^, which is used in the OpenCV camera calibration module^17^. In this work, the target was made of stainless steel, as it is the most common and reliable material used in clinics for medical devices. This material is freely available, not expensive, robust, and easy to sterilize. Other materials could potentially be used for the calibration target, such as aluminum or iodized metals, but this is the scope of future work.


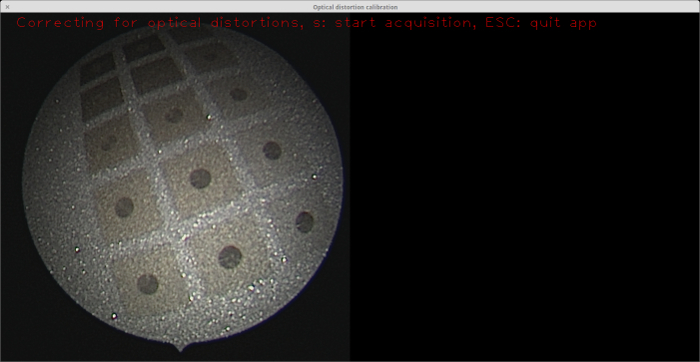
**Figure 7: Stainless steel plate featuring a palette of different background colors etched with the laser. **Practical experiments were conducted in conjunction with the OpenCV feature detection module to determine which background color gives the optimal result in terms of blob-to-background contrast^23^. The endoscope view on the left shows the plate. The moderate background colors (*i.e.,* those other that the darkest and lightest ones) in this palette yield better blob detection. Please click here to view a larger version of this figure.

One of the advantages of this work is that performing a calibration using the fabricated target takes 2-3 min. Most of the effort goes to manually stabilizing the endoscope to obtain decent views of the calibration pattern. Using a custom-built endoscope holder could eliminate the need for manual stabilization, which in turn could significantly shorten calibration time.


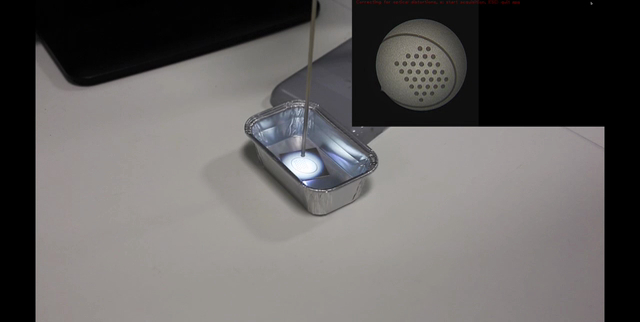
 **Video 1: Video showing how optical distortion calibration can be performed using the developed calibration target together with the endocal software.** Please click here to view this video. (Right-click to download.)

An advantage of our work compared to the work of Wengert *et al.*^15^ is that the OpenCV camera calibration module^17^ can be used as is for calibration, without requiring any modification or custom parameterization. Because OpenCV is a well-established and well-maintained software package and is very popular in the computer vision community, using it eliminates the need for writing and maintaining custom software. For the reader's convenience, a compact GUI application is provided^16^, which the reader can easily install and use to test new calibration targets. One disadvantage of our method compared to Wengert *et al.*^15^ is that their method is more robust to occlusions of the pattern, as it does not require the detection of all blobs.

Initially, a calibration target with a checkerboard pattern was fabricated for this work. However, this type of calibration target proved to be unsuitable in experiments due to the difficulty of detecting the corners of the checkerboard squares. Corner detection relies on histogram-based image binarization (see the OpenCV source code^24^). This implies the need for a clear color contrast between the dark and light squares, which could not be guaranteed with our checkerboard pattern, partially due to specular reflections, like the ones shown in **Figure 6**. Such specular reflections are present even after background etching; however, the detection of the circles seems to be less sensitive to this shortcoming.

In the current setup, only perpendicular views of the calibration target allow for successful blob detection. This is due to the specular reflections from the target surface hampering blob detection at oblique angles. We are working to further improve the target so as to allow for the acquisition of views at a wider range of angles, which could potentially improve the quality of performed calibrations^20^.

In the real-time placental mosaicing pipeline that was previously proposed^11^, the computation of the transformation that maps image pairs relies on the successful detection and grouping of features. Optical distortions, on the other hand, cause a group of features with a rigid geometry to appear different across images. As a consequence, this difference leads to inaccuracies in the computed transformations, which cause drifts in the resulting image mosaics. Because the most prominent optical distortions are present towards the edges, endoscopic images are currently cropped to their innermost regions. A good correction for optical distortions would potentially allow for the incorporation of a larger part of each image into the mosaicing process. The advantage of this method is two-fold. First, it would increase the number of detected features in each image, potentially improving the computation of the image transformations. Second, it would allow for the whole target anatomical surface to be reconstructed in a shorter time.

## Disclosures

The authors have nothing to disclose.
